# Editorial: New Frontiers in *Wolbachia* biology 2025

**DOI:** 10.3389/fmicb.2026.1980203

**Published:** 2026-09-30

**Authors:** Takema Fukatsu, Mark J. Taylor, Toshiyuki Harumoto, Yuval Gottlieb, Steve Perlman, Zhiyong Xi, Amelia R. I. Lindsey, Susumu Katsuma

**Affiliations:** 1 Molecular Biosystems Research Institute, National Institute of Advanced Industrial Science and Technology, Tsukuba, Japan; 2 Graduate School of Life and Environmental Sciences, University of Tsukuba, Tsukuba, Japan; 3 Department of Biological Sciences, Graduate School of Science, The University of Tokyo, Tokyo, Japan; 4 Department of Tropical Disease Biology, Liverpool School of Tropical Medicine, Liverpool, United Kingdom; 5 Life Science Center for Survival Dynamics, Tsukuba Advanced Research Alliance (TARA), University of Tsukuba, Tsukuba, Japan; 6 Koert School of Veterinary Medicine, Robert H. Smith Faculty of Agriculture, Food and Environment, The Hebrew University of Jerusalem, Rehovot, Israel; 7 Department of Biology and Centre for Forest Biology, University of Victoria, Victoria, BC, Canada; 8 Department of Microbiology, Genetics, and Immunology, Michigan State University, East Lansing, MI, United States; 9 Department of Entomology, University of Minnesota, Saint Paul, MN, United States; 10 Department of Agricultural and Environmental Biology, Graduate School of Agricultural and Life Sciences, The University of Tokyo, Tokyo, Japan

**Keywords:** diversity, insect, nematode, symbiont, *Wolbachia*

*Wolbachia* is among the most widespread and successful intracellular symbionts in nature, infecting an extraordinary diversity of arthropods as well as numerous nematodes worldwide (Werren et al., 2008; Serbus et al., 2008). Its remarkable ability to manipulate host reproduction, influence host fitness, interact with pathogens, and establish intimate cellular associations has made *Wolbachia* a leading model for investigating host–microbe interactions across multiple biological scales. At the same time, applications of *Wolbachia* have expanded rapidly, particularly for controlling vector-borne diseases and treating filarial infections (Ross et al., 2019; Johnston et al., 2021).

Despite major advances over recent decades, many fundamental questions remain unresolved. How do intracellular symbionts manipulate host physiology at the molecular level? How extensive is the natural diversity of *Wolbachia* and related symbiotic systems? How can insights from symbiosis research be translated into solutions to challenges in medicine, agriculture, and environmental management? Since the first *Wolbachia* Conference held in Crete, Greece, in 2000, the biennial conference series has provided an invaluable forum for communication and collaboration among *Wolbachia* researchers worldwide (Fukatsu et al., 2024). In conjunction with the 12th *Wolbachia* Conference held in Okinawa, Japan, from 13 to 19 April 2025, the Research Topic “*New Frontiers in Wolbachia biology 2025*” was launched to showcase recent advances in this rapidly developing field. The resulting Research Topic comprises 11 research articles spanning three major directions: elucidating the mechanisms of host–symbiont interactions, exploring evolutionary and ecological complexity, and translating symbiosis research into practical applications.

One prominent theme emerging from this Research Topic concerns the molecular and cellular mechanisms underlying phenotypic trade-offs in host–symbiont interactions. Dou and Li combined transcriptomic and small-RNA sequencing analyses in *Drosophila melanogaster* to investigate the molecular basis of differences between offspring derived from cytoplasmic incompatibility crosses and their parental lines. Male offspring showed marked activation of immune- and detoxification-related pathways, accompanied by broad suppression of pathways involved in spermatogenesis, energy metabolism, and small-RNA regulation. These patterns suggest an immune–reproduction trade-off associated with intergenerational disruption of host small-RNA regulatory networks. Robinson et al. revealed substantial fitness costs associated with feminizing *Wolbachia* infections in the spider *Mermessus fradeorum*, providing new insight into the evolutionary constraints that shape the distribution of reproductive manipulation phenotypes. Umanzor et al. showed that the facultative intracellular symbiont *Lariskella*, an underexplored endosymbiont associated with diverse insects and other arthropods (Hagenbeek et al., 2026), induces cytoplasmic incompatibility while conferring little detectable fitness benefit on the leaf-footed bug *Leptoglossus zonatus*.

Methodological innovation constitutes another prominent theme of this Research Topic. Sinha et al. applied Cappable-seq to characterize density-dependent transcriptional responses of *Wolbachia* while simultaneously detecting active RNA viruses in a mosquito cell line. Behrmann et al. developed an improved RNA-seq workflow that substantially enriches *Wolbachia* transcripts from mosquito cells, providing a useful protocol for future studies on intracellular bacterial gene expression.

Another major theme is the diversity and ecological complexity of *Wolbachia*-associated systems. Kaur and Brown expanded the known host range of *Wolbachia* by discovering a novel infection in the plant-parasitic nematode genus *Heterodera*. Phylogenetic analyses placed this bacterium close to *Wolbachia* strains previously reported from other plant-parasitic nematodes (Haegeman et al., 2009; Brown et al., 2016). Itokawa et al. found that *Wolbachia* frequently co-infects the sand fly *Sergentomyia squamirostris* with *Tisiphia*, a recently recognized and still underexplored endosymbiont that is widespread among invertebrates (Davison et al., 2022). Luo et al. investigated the diversity and evolutionary dynamics of WO bacteriophages in crickets, reinforcing the view that phages are important drivers of *Wolbachia* diversification (Kent and Bordenstein, 2010). Ferreira et al. demonstrated that viral community composition and abundance in the spider mite *Tetranychus truncatus* vary substantially under diverse biotic and abiotic conditions, including acaricide exposure, elevated temperature, and infection with *Wolbachia* or *Spiroplasma*. Their findings reveal a previously underappreciated dimension of viral dynamics within host–microbe symbiotic systems.

Finally, several studies highlight the potential relevance of symbiosis research to agriculture and medicine. Ohata and Tagami examined how agrochemicals containing antibiotics affect endosymbiont prevalence and population dynamics in an experimental agricultural ecosystem. Their results demonstrate that human-mediated perturbations can reshape symbiotic associations within ecological communities and underscore the potential unintended consequences of agricultural practices. Yousef et al. identified autophagy as a critical determinant of anti-*Wolbachia* drug activity, thereby linking fundamental cell biology to the development of strategies for controlling filarial diseases.

Taken together, the articles assembled in the Research Topic “*New Frontiers in Wolbachia biology 2025*” showcase the remarkable breadth of contemporary research on *Wolbachia* and related symbioses. Collectively, they illustrate a field moving rapidly beyond the study of a single fascinating intracellular bacterium. *Wolbachia* now serves as a gateway to broader questions about how symbiotic interactions shape biological systems across scales—from cellular networks and genomes to populations, communities, and ecosystems. As methodological capabilities expand and interdisciplinary collaborations deepen, *Wolbachia* research will continue to advance our understanding of host–microbe interactions while contributing to innovative solutions in human health, agriculture, and biodiversity conservation.

## References

[B1] Brown A. M. V. Wasala S. K. Howe D. K. Peetz A. B. Zasada I. A. Denver D. R. (2016). Genomic evidence for plant-parasitic nematodes as the earliest *Wolbachia* hosts. Sci. Rep. 6:34955. doi: 10.1038/srep34955 27734894 PMC5062116

[B2] Davison H. R. Pilgrim J. Wybouw N. Parker J. Pirro S. Hunter-Barnett S. . (2022). Genomic diversity across the *Rickettsia* and ‘*Candidatus* Megaira' genera and proposal of genus status for the Torix group. Nat. Commun. 13:2630. doi: 10.1038/s41467-022-30385-6 35551207 PMC9098888

[B3] Fukatsu T. Gottlieb Y. Tsiamis G. McGraw E. Perlman S. Bouchon D. . (2024). Editorial: Frontiers in *Wolbachia* biology 2023. Front. Microbiol. 15:1513314. doi: 10.3389/fmicb.2024.1513314 39588098 PMC11587352

[B4] Haegeman A. Vanholme B. Jacob J. Vandekerckhove T. T. M. Claeys M. Borgonie G. . (2009). An endosymbiotic bacterium in a plant-parasitic nematode: member of a new *Wolbachia* supergroup. Int. J. Parasitol. 39, 1045–1054. doi: 10.1016/j.ijpara.2009.01.006 19504759

[B5] Hagenbeek A. Masukagami Y. Palanichamy P. Husnik F. (2026). Genome-resolved metagenomics reveals unexpected diversity and host range of *Candidatus* Lariskella (Rickettsiales: Midichloriaceae). BMC Genomics 27:559. doi: 10.1186/s12864-026-12881-x 42069539 PMC13289439

[B6] Johnston K. L. Hong W. D. Turner J. D. O'Neill P. M. Ward S. A. Taylor M. J. (2021). Anti-*Wolbachia* drugs for filariasis. Trends Parasitol. 37, 1068–1081. doi: 10.1016/j.pt.2021.06.004 34229954

[B7] Kent B. N. Bordenstein S. R. (2010). Phage WO of *Wolbachia*: lambda of the endosymbiont world. Trends Microbiol. 18, 173–181. doi: 10.1016/j.tim.2009.12.011 20083406 PMC2862486

[B8] Ross P. A. Turelli M. Hoffmann A. A. (2019). Evolutionary ecology of *Wolbachia* releases for disease control. Annu. Rev. Genet. 53, 93–116. doi: 10.1146/annurev-genet-112618-043609 31505135 PMC6944334

[B9] Serbus L. R. Casper-Lindley C. Landmann F. Sullivan W. (2008). The genetics and cell biology of *Wolbachia*-host interactions. Annu. Rev. Genet. 42, 683–707. doi: 10.1146/annurev.genet.41.110306.130354 18713031

[B10] Werren J. H. Baldo L. Clark M. E. (2008). *Wolbachia*: master manipulators of invertebrate biology. Nat. Rev. Microbiol. 6, 741–751. doi: 10.1038/nrmicro1969 18794912

